# Self-reported adverse reactions in 4337 healthcare workers immunizations against novel H1N1 influenza

**DOI:** 10.1186/1756-0500-4-297

**Published:** 2011-08-17

**Authors:** Harald Bias, David Quarcoo, Claus Meier-Wronski, Sabine Wicker, Joachim Seybold, Albert Nienhaus, David A Groneberg, Andres de Roux

**Affiliations:** 1 Occupational Medical Service, Charité - Universitätsmedizin Berlin, Free University and Humboldt University, D-14195 Berlin, Germany; 2 Institute of Occupational Medicine, Social Medicine and Environmental Medicine, Johann Wolfgang Goethe-University, Theodor-Stern-Kai 7, 60590 Frankfurt am Main, Germany; 3 Institute of Occupational Medicine, Charité - Universitätsmedizin Berlin, Free University and Humboldt University, D-14195 Berlin, Germany; 4 Occupational Health Service, Johann Wolfgang Goethe-University, Theodor-Stern-Kai 7, 60590 Frankfurt am Main, Germany; 5 Medical Directorate, Charité - Universitätsmedizin Berlin, Free University and Humboldt University, D-14195 Berlin, Germany; 6 Institution for Statutory Accident Insurance and Prevention in the Health and Welfare Services, Department of Occupational Health Research, Pappelallee 35/37, Hamburg 22089, Germany

**Keywords:** adverse reaction, healthcare worker, immunization, novel H1N1 influenza

## Abstract

**Purpose:**

The use of the 2009 H1N1 vaccine has generated much debate concerning safety issues among the general population and physicians. It was questioned if this is a safe vaccine. Therefore, we investigated the safety of an inactivated monovalent H1N1 pandemic influenza vaccine

**Methods:**

We focused on the H1N1 pandemic influenza vaccine Pandemrix^® ^and applied a self reporting questionnaire in a population of healthcare workers (HCWs) and medical students at a major university hospital.

**Results:**

In total, 4337 individuals were vaccinated, consisting of 3808 HCWs and 529 medical students. The vaccination rate of the employees was higher than 40%. The majority of individuals were vaccinated in November 2009. In total, 291 of the 4337 vaccinations were reported to lead to one or more adverse reactions (6.7%). Local reactions were reported in 3.8%, myalgia and arthralgia in 3.7%, fatigue in 3.7%, headache in 3.1%.

**Conclusions:**

Our data together with available data from several national and international institutions points to a safe pandemic influenza vaccine.

## Introduction

Various infectious diseases play a major role in occupational health. Next to classical diseases such as tuberculosis [1], new diseases including SARS [2-4] or the novel influenza A H1N1/2009 virus [5,6] are endangering occupational health in the last years. The novel influenza A H1N1/2009 virus was first identified in Mexico and led to pandemic warning of the WHO in June 2009 [7].

Novel influenza A H1N1/2009 virus is often called "swine flu" and represents a result of the reassortment of different influenza viruses [8,9]. It was reported that the hemagglutinin (HA) gene of A H1N1/2009 was similar to that of swine flu viruses which are present in United States pigs since the year 1999. By contrast, the matrix protein (M) and neuraminidase (NA) genes are found in European swine flu isolates. Phylogenetic analysis of the pandemic H1N1/2009 virus shows that six genome segments stem from a triple-reassortant virus circulating in North American swine, seeded from human, avian and classical swine lineages.

Concerning the pandemic, a first outbreak of Influenza-like illness occurred in Mexico and the USA in April 2009 and the US Centers for Disease Control and Prevention reported seven cases of novel A/H1N1 influenza by this time.

By April 24 the WHO issued a health advisory on the outbreak of "influenza like illness in the United States and Mexico". Despite measures by the Mexican government against the spread of the virus, the number of confirmed cases raised to 2,099 by May 7 2009 [10]. One month later, on June 11, 2009, the WHO officially declared a H1N1 pandemic [11]. This was the first pandemic since the 1968 Hong Kong flu pandemic. The WHO alert level was lifted to phase 6.

Different vaccines were generated: In autumn 2009, GlaxoSmithKline produced the vaccine Pandemrix^® ^[12]. Other vaccines were Focetria^®^, made by Novartis and Celvapan^®^, made by Baxter.

The safety of influenza vaccine is in the focus of research since many years [13-17]. Due to a special debate on the safety of the pandemic influenza vaccines [18], it was the objective of the present study to analyse the safety using a self reporting questionnaire approach in the acute event of a pandemic and a novel vaccine which was debated for its safety by the general population and healthcare worders (HCWs). We chose a population of healthcare workers (HCWs) and medical students after vaccination with Pandemrix^® ^since according to German federal recommendations HCWs had a top priority for vaccination [19].

## Methods

### Vaccine

After the declaration of the pandemic, the German federal commission for vaccination (STIKO) recommended the vaccination for HCWs. The local occupational health service of the University Hospital Berlin Charité was supplied with 6000 Pandemrix^® ^doses on October 26, 2009.

The active antigen of Pandemrix^® ^derived from the A/California/7/2009 (H1N1). Pandemrix^® ^also contains an immunologic adjuvant AS03 which consists of DL-α-tocopherol (vitamin E), squalene and polysorbate 80. Thiomersal (thimerosal) is added as a preservative. Other components are formaldehyde, sodium deoxycholate, and sucrose [20]. The vaccine is generated in hen's eggs and therefore also contains traces of egg proteins. A single dose of the vaccine was injected by occupational physicians with the recommended dosage and vaccination procedure.

### Sample selection method

In the situation of a pandemic and the acute supply of a novel vaccine which included the immunologic adjuvant AS03 and thiomersal (thimerosal), and a public debate about the safety of this vaccine, the sample selection method based on the assessment of all individuals that were vaccinated after informed consent at the occupational medicine centre of Germany largest university hospital. No specific further sample selection methods were applied.

### Population

In total, 3808 HCWs and 529 medical students were vaccinated with Pandemrix^® ^after informed consent.

### Period

The observed vaccination period began on October 26, 2009 and ended at December 30, 2009. All individuals that were vaccinated after informed consent.

### Self reporting questionnaire

Applying a cross-sectional study design, a self reporting questionnaire (Tab. 1) was used that consisted of questions relating the time of the vaccination, the start of symptoms, the duration of symptoms, the consultation of a physician and the incapacity for work with duration. The symptoms included in the questionnaire are presented in Appendix 1. The self reporting questionnaires were received until 2010-01-31.

## Results

### Population

**Figure 1:**
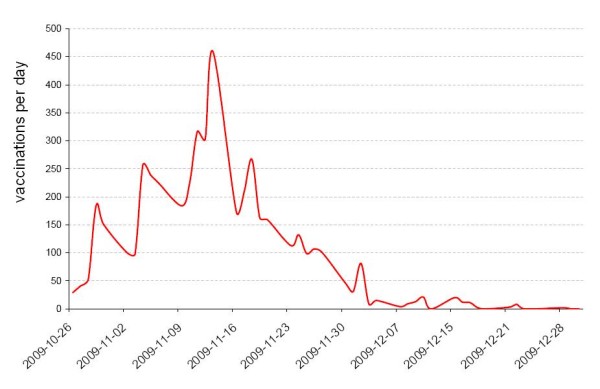
**Daily numbers of vaccinations in the period from November to December 2009**.

In total, 3808 HCWs and 529 medical students were vaccinated. The peak of vaccinations was November 13 2009 with 459 vaccinations at one day (Figure 1).

### Self reported adverse reactions

**Figure 2:**
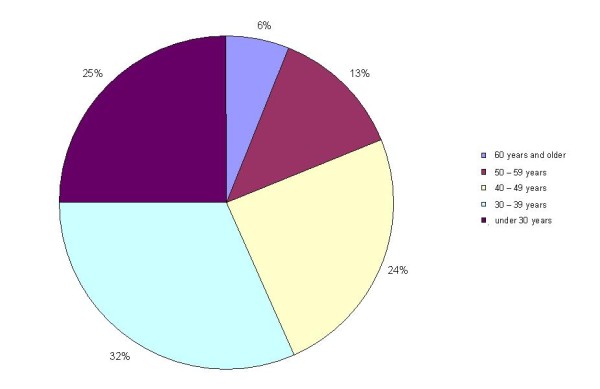
**Distribution of age of study participants**.

Overall, 291 of the 4337 vaccinated individuals returned the questionnaire and reported adverse reactions. This is a rate of 6.7%. The majority of reported adverse reactions was found in the age between 30 and 39 years. (Figure 2)

**Figure 3:**
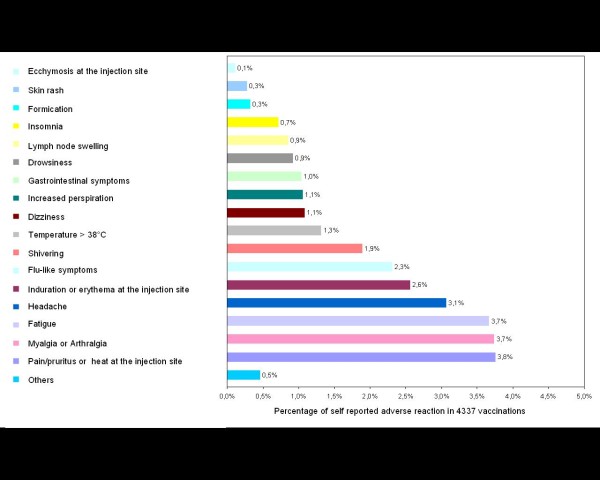
**Percentage of self reported adverse reactions**.

The most frequently reported local site reactions were: local pain/pruritus or the sensacion of heat at the injection site in 3.8% out of the 4337 vaccinations, myalgia or arthralgia in 3.7%, induration or erythema at the injection site in 2.6%, lymph node swelling in 0.9%, skin rash in 0.3% and ecchymosis at the injection site in 0.1% (Figure 3).

The presence of systemic adverse reactions were reported as follows: fatigue in 3.7%, headache in 3.1%, flu-like symptoms in 2.3%, shivering in 1.9%, temperature > 38°C in 1.3%, dizziness in 1.1%, increased perspiration in 1.1%, gastrointestinal symptoms in 1.0%, drowsiness in 0.9%, , insomnia in 0.7%, formication in 0.3%, Further some severe reportable adverse reactions were observed (0.5%, Figure 3) as one case of facial nerve paralysis, one case of rheumatoid arthritic symptoms and one case of skin alteration which was reported to the local health authorities and the Paul-Ehrlich-Institute.

### Duration of symptoms

The mean duration of symptoms lasted 3.5 days, the maximal duration of symptoms was reported with 40 days.

### Leave of absence

**Figure 4:**
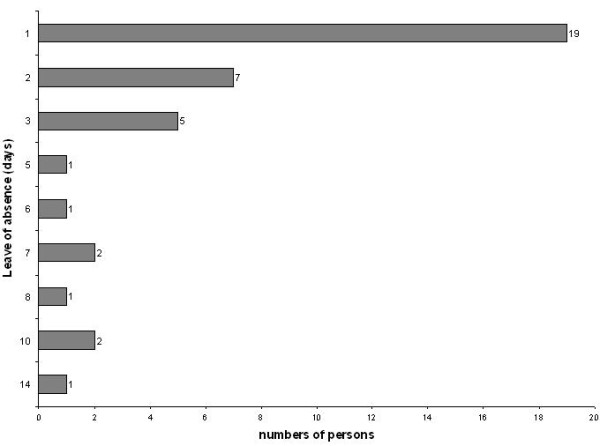
**Leave of absence in days due to the reported adverse reactions**.

Overall, 42 HCWs (0.97%) were not able to work due to the adverse reactions (Figure 4). The resulting leave of absence was 2.7 days in mean with a maximum absence of 14 days in one case. In total, there were 115 days of absence recorded.

## Discussion

Personal protective equipment is important to prevent transmission of novel A/H1N1 as stated earlier by Shine et al [21]. However, vaccination is the most effective means of preventing influenza transmission and associated morbidity and mortality. It is most important to realize that an effective measure against a pandemic is to have vaccinated and well-informed health care workers.

Unfortunately, the A/H1N1 vaccination coverage was extremely affected by an ongoing public discussion about potential side effects. Therefore, we analysed self reporting questionnaires concerning adverse reactions in 4337 HCWs and medical students.

Of course, this study - that was initiated in the acute event of a pandemic and a safety discussion - has a variety of limitations. Apart from the paucity of demographic data, self reporting questionnaires are largely limited since there may be a number of individuals who do not return the questionnaire despite adverse reaction manifestation. However, there was a need to assess potential adverse reactions since the general population and the HCWs asked for data about the new vaccine. Therefore, we decided to undertake a self reporting study despite that fact that the extent of underreporting of side effects can not be examined precisely in the chosen design. It is noteworthy that with our self reporting system we found a rate of 6.7% (291 of 4337 vaccinations). This frequency differs slightly from data from other studies but points to a safe vaccine in terms of acute adverse reactions. A prospective, randomised study with 178 participants by Vajo et al. concluded that all adverse events were rare, mild, and transient. Using the vaccine Fluval P, the most frequent reactions in this study were pain at injection site (eight cases) and fatigue for 1-2 days after vaccination (three cases) [22]. Concerning the vaccination rate we can report a rate of 4337 of about 10 000 employees of the hospital. This is a vaccination rate of over 40%. In a parallel study in Frankfurt/Main, the influenza vaccination rates of the HCWs of the University Hospital Frankfurt were measured. In this study, we were also able to show that the 2009 vaccination rate (seasonal influenza [40.5%], swine flu [36.3%]) was better than the average annual uptake of influenza vaccine in the German health care system (approximately 22% for seasonal and 15% for swine flu) [23].

In meantime, a number of studies were published that also addressed safety issues of the H1N1 vaccination in healthcare workers [24-26]. I.e. an inactivated, split-virus, unadjuvanted AH1pdm vaccine, manufactured in Japan, was given to HCWs from October 19, 2009. A retrospective cohort study was conducted and and severe adverse events were rare [24]. A recent study using monovalent vaccination (Panenza; Sanofi Pasteur, Val de Reuil Cedex, France) among HCWs in a university hospital setting in Thailand also reported a low rate of side effects. The most common adverse reaction was fatigue/uncomfortable feeling (24%) [25].

## Conclusions

It can be summarized that our data points to a safe pandemic influenza vaccine in our 4337 vaccinations. It needs to be taken into account that the use of self reporting questionnaires leads to differing results concerning the frequency of adverse reactions, of course. Therefore, this mode of reporting should be interpreted cautiously and only applied in the acute event of pandemics/novel vaccines that are administered without the usual safety testings.

## Competing interests

The authors declare that they have no competing interests.

## Authors' contributions

HB carried out the study, participated in the analysis and drafted the manuscript. DQ participated in the analyzing and drafting process. CM-W and SW participated in the pilot study design. JS and AN participated in the design of the study and the statistical analysis. DAG and AdR conceived of the study, and participated in its design and coordination and helped to draft the manuscript. All authors read and approved the final manuscript.

## Appendix 1: Symptoms included in the questionnaire

Pain/pruritus or heat at the injection site

Induration or erythema at the injection site

Ecchymosis at the injection site

Lymph node swelling

Flu-like symptoms

Temperature > 38°C

Shivering

Headache

Fatigue

Myalgia or Arthralgia

Gastrointestinal symptoms

Insomnia

Formication (pins and needles)

Drowsiness

Dizziness

Increased perspiration (sweating)

Skin rash

Others (please specify)
